# Sleep disorders in chronic pain and its neurochemical mechanisms: a narrative review

**DOI:** 10.3389/fpsyt.2023.1157790

**Published:** 2023-06-01

**Authors:** Lan Duo, Xintong Yu, Ruihan Hu, Xiping Duan, Jia Zhou, Ke Wang

**Affiliations:** Acupuncture Anesthesia Clinical Research Institute, Yueyang Hospital of Integrated Traditional Chinese and Western Medicine, Shanghai University of Traditional Chinese Medicine, Shanghai, China

**Keywords:** chronic pain, sleep disorders, pain and sleep comorbidity, pain treatment, sleep disorder treatment

## Abstract

Chronic pain (CP) is a prevalent problem, and more than half of patients with CP have sleep disorders. CP comorbidity with sleep disorders imposes immense suffering and seriously affects the patient’s quality of life, which is a challenging issue encountered by clinicians. Although the reciprocal interactions between pain and sleep have been studied to some degree, there is still a lack of awareness and comprehensive description of CP comorbidity with sleep disorders. In this narrative review article, we summarize the current knowledge about the present estimates of the prevalence of comorbid sleep disorders in CP patients, sleep detection methods, sleep characterization in CP, and the effect of sleep disorders on CP and current therapies. We also summarize current knowledge of the neurochemical mechanisms of CP comorbidity with sleep disorders. In conclusion, insufficient attention has been paid to the role of sleep disorders in CP patients, and CP patients should be screened for sleep disorders in the clinic. Special attention should be given to a possible risk of drug–drug interaction when using two types of drugs targeting pain and sleep simultaneously. The current insight into the neurobiological mechanisms underlying CP comorbidity with sleep disorders is still rather limited.

## 1. Introduction

Chronic pain (CP) refers to pain that persists or recurs for more than 3 months, which was listed as an independent disease for the first time in the International Classification of Diseases (ICD-11) revised by the World Health Organization (WHO) in 2018 (1). About 13%–25% of the general population is affected by CP, which is a critical clinical issue and one of the major causes of global impairment (2, 3). Numerous CP patients suffer from poor quality of life, including sleep issues, anxiety, and depression (4–6). According to a survey report in the United States (US), the annual economic loss caused by CP is approximately 560–635 billion US dollars (7).

Sleep is an important physiologic process to maintain homeostasis and function of the body (8). Over 65% of CP patients report having trouble sleeping, making it one of their main complaints (9). Sleep problems include difficulty falling asleep, sleep insufficiency, and low sleep quality, which can lead to a wide range of physical and mental problems (10–12). There is a direct relationship between the degree of sleep issues and the intensity of pain (13). For instance, fibromyalgia can significantly alter the architecture of sleep (14–16). Similarly, poor sleep has negative impacts on CP. According to the Trøndelag Health (HUNT) study, which monitored participants for up to 22 years, people who reported having insomnia symptoms for more than 10 years in combination with short sleep were at an especially high risk of experiencing recurrent spinal pain, and improvement in insomnia symptoms was associated with a favorable prognosis (17). A systematic review and meta-analysis also found that sleep disturbances and disorders were significantly related to chronic postsurgical pain (18). Additionally, people with sleep issues have higher levels of pain-related anxiety, medicine use, and self-reported diseases compared to pain patients who sleep properly (13). Thus, it is clear that CP and sleep disorders are frequently thought to interact with each other. The relevance of the mutual interaction between the two has been gradually realized as more in-depth studies on the comorbidity of CP and sleep problems have been conducted, which has also become a hot topic in pain research. As a result, therapeutic care becomes more intricate and difficult when a sleep disturbance develops in a patient with CP. Given the importance of sleep disorder in the development and unfavorable prognosis of CP, research has begun to increase in recent years to explore the basic neurochemical mechanisms underlying this reciprocal relationship. We examined and condensed an overview of the development of this problem into key areas in this article: clinical presentation and sleep monitoring, therapy and prognosis, and underlying mechanism.

## 2. Clinical research on the relationship between chronic pain and sleep

### 2.1. Epidemiological study of chronic pain complicated with sleep disturbance

Sleep disorders are also a major public health problem that plagues human physical and mental health (19). According to research data, 27% of people in the world have sleep disorders (20). In numerous experimental sleep deprivation models, limiting or interrupting sleep for a day or a few days can cause sleep disorders such as insufficient sleep time and poor sleep quality, which can result in hyperalgesia or spontaneous pain, exacerbating chronic pain (21). According to studies, 10%–15% of the general population suffers from insomnia (22). Sleep and pain are two vital physiological functions that interact with each other and have an impact on one another in humans. According to research, at least 40% of people with insomnia also have CP, and 50%–88% of CP patients have sleep difficulties (20). Diagnosed according to the Diagnostic and Statistical Manual of Mental Disorders (DSM) criteria, insomnia is more common in the CP community than in the general population, with a frequency of 24%–32% (22). Meanwhile, a recent meta-analysis has shown that individuals with CP experience significant sleep disturbances, particularly with respect to sleep initiation and maintenance (23). This study has also found that the pooled prevalence of sleep disorders in CP was 44%, with insomnia (72%), restless legs syndrome (32%), and obstructive sleep apnea (32%) being the most common diagnoses (23).

### 2.2. Evaluation and tracking of sleep quality

Various sleep indicators can be used to assess CP patients’ sleep quality or duration, including sleep onset time, wakefulness after sleep onset (WASO), sleep onset latency (SOL), sleep efficiency (SE), and total sleep time (TST). There are both subjective and objective methods to assess sleep quality. In terms of objective methods for monitoring sleep, polysomnography (PSG) and actigraphy have high reliability in obtaining information on sleep parameters. PSG is the gold standard method for analyzing sleep quality, using many sensors and electronics; however, it requires a cumbersome, complex setup of electronic sensors and needs to be performed in a laboratory under the control of trained technicians. These requirements may disrupt natural sleep patterns, and one-night sleep data are often insufficient to represent normal sleep behavior. Therefore, it cannot be used frequently in clinical practice due to the disadvantages of economic cost and time costs (24). Nowadays, actigraphy is the most extensively used device to assess sleep quality at home. Actigraphy is a relatively inexpensive and non-invasive method of assessing sleep–wake rhythms over long periods, from days to months (25). It has certain clinical value for sleep disorders accompanied by sleep rhythm disturbance, emotional disorder and body movement abnormality (26). Compared with PSG, actigraphy has limited accuracy in detecting wakefulness during sleep episodes and does not provide information on sleep architecture (27). In terms of subjective methods, many self-report questionnaires have been developed to assess sleep, such as Pittsburgh Sleep Quality Index (PSQI), Sleep Severity Scale (AIS), Sleep Severity Index (ISI), Mini Sleep Questionnaire (MSQ), Jenkins Sleep Scale (JSS), Leeds Sleep Assessment Questionnaire (LSEQ), and Epworth Sleep Scale (ESS) (24). These questionnaires can record sleep quality by estimating subjective sleep quality, sleep latency, sleep duration, habitual sleep efficiency, sleep disturbance, use of sleep medications, and daytime dysfunction as indicators. Among these questionnaires, the PSQI is the most commonly used measure of subjective self-report sleep quality, which is considered an accepted reference or gold standard for self-perceived sleep quality (28, 29).

### 2.3. Sleep disturbances characterization in chronic pain

Sleep disturbances include reduced SE and altered sleep architecture, which occur in patients with CP (30). CP patients have a decrease in TST and SE and an increase in SOL, WASO, and a number of awakenings, indicating that patients with CP have less sleep time, take longer to fall asleep, and spend more time awake (23, 31). SE is the ratio of total sleep time to total time in bed. Many studies have found that lower SE has a strong association with next-day pain intensity in CP patients (32, 33). A review has found that there is high heterogeneity in sleep architecture according to individual study reports (31). However, a recent meta-analysis has found that CP patients spent more time in the first stage of sleep during the non-rapid eye movement (NREM) and experienced more sleep fragmentation (23). Sleep fragmentation refers to shortened sleep bouts and frequent transitions between sleep and wake states. Patients with CP had a more frequent transition from sleep to wakefulness (23). Sleep fragmentation could impair endogenous pain-inhibitory function in both healthy adults and CP patients (34, 35). One animal experiment has also confirmed that sleep fragmentation combined with musculoskeletal sensitization could exacerbate mechanical hypersensitivity; increase the number of sleep–wake state transitions during the light and dark periods; change NREM sleep, rapid eye movement sleep, and wakefulness; and alter delta power during NREM sleep (36).

### 2.4. Consequences of chronic pain combined with sleep disorders

Poor sleep is a key factor in the development and maintenance of CP (9). Sleep deprivation can increase subjective pain intensity and worsen peripheral/central pain sensitization in healthy individuals (37). Sleep disorders significantly increase the risk for reduced pain tolerance, and sleep extension increases pain tolerance in healthy individuals (38, 39). Sleep disorders are also one of the most common triggers of migraine (40). Patients with both CP and sleep disturbances have greater pain severity, longer duration of pain, greater disability, are less physically active than those without sleep disturbances, and are more likely to have concurrent depression, catastrophizing, anxiety, and suicidal ideation (22). For example, restless sleep can increase depressive symptoms and fatigue among individuals with knee osteoarthritis (41). The coexistence of insomnia and chronic musculoskeletal pain results in greater pain intensity and alterations in sleep homeostasis (42). Among patients with neuropathic pain, those with poor sleep quality experience more pain, more severe depressive states, and worse quality of life than patients with good sleep quality, with a positive correlation between sleep quality and emotional state (43). In patients with musculoskeletal disorders, poorer sleep quality is associated with higher pain levels, and a high frequency of poor sleep quality is more prevalent in females (44). Another study has also found that sleep deficiency, particularly insomnia and poor sleep quality, might modify the effectiveness of psychological treatments for CP (45). If participants report better sleep quality, they will have less pain and better self-rated health on the following day (46). Improving sleep quality is an important approach to reduce the CP burden (47). Another systematic scoping review has suggested that sleep disturbances, and sleep disorders were associated with worse pain outcomes and treatment-induced sleep improvements ameliorated pain outcomes among veterans with CP (48). Even short-term improvements in sleep can predict long-term improvements in CP and fatigue in older adults with osteoarthritis (49, 50). On the other hand, a cross-sectional study has found that CP can directly affect sleep quality, and poor sleep quality can further lead to depression (51). The aforementioned results indicate that the accurate assessment and timely treatment of sleep problems are important treatment modalities for pain management.

### 2.5. Effects of therapy on sleep disorder of patients with chronic pain

Due to the significant sleep-pain interactions, it has been suggested that multidisciplinary treatment is required to manage CP. Currently, treatments for sleep disorder of patients with CP include pharmacotherapy and non-pharmacological therapy.

#### 2.5.1. Medications

In terms of pharmacotherapy, two aspects are particularly important to consider. The first aspect is that medications used to treat pain or sleep can also have direct effects on sleep or pain, respectively. The second aspect is that multiple drugs are frequently used in clinical practice to target sleep disorders and CP simultaneously, which might increase the risk of drug interactions. This topic is discussed in detail in a review article by Herrero et al. (52). Here, therefore, only a brief summary is provided.

##### 2.5.1.1. The effect of drugs used in pain management on sleep

###### 2.5.1.1.1. Opioids

Opioids are widely used for the treatment of chronic pain. Recently, a study has found that current and previous chronic users of opioids had differed significantly from the opioid-naïve regarding sleep quality, sleep duration, sleep disturbances, and daytime dysfunction after controlling for sleep medications in CP patients, and opioid-naïve participants had better sleep quality (53, 54). Another study has also found that opioid use was associated with a 30% increased risk of poor-quality sleep and an approximately 15% increased risk for short sleep duration in older adults with CP (53, 54). In addition, chronic opioid use increases the risk of sleep apnea and sleep-disordered breathing (55–57) and may lead to an increased risk of death in patients with chronic non-cancer pain (55). A prospective cohort observational study has suggested that chronic non-cancer pain patients with *OPRM1* 118-GG genotype were more susceptible to an increase in sleep problems and worsening sleep patterns while taking opioids (58).

###### 2.5.1.1.2. Cannabinoids

In response to the opioid epidemic, medical cannabinoids are increasingly used to manage chronic pain (59, 60). Multiple systematic reviews and meta-analyses have shown that medical cannabis and cannabinoids may improve impaired sleep in patients with CP, including sleep quality, insomnia, obstructive sleep apnea, REM sleep behavior disorder, and excessive daytime sleepiness (61–63).

###### 2.5.1.1.3. Antiseizure medications

Gabapentin and pregabalin are both gamma-aminobutyric acid (GABA) analogs for the treatment of neuropathic pain and have positive effects on sleep disturbances in neuropathic pain (64). A meta-analysis has demonstrated that longer-duration gabapentin treatment could significantly improve sleep health in patients with chronic neuropathic pain (65). Pregabalin has also demonstrated efficacy for sleep improvement by reducing sleep interference scores and improving the mean sleep scores in patients with neuropathic pain (66, 67).

###### 2.5.1.1.4. Tricyclic antidepressants

Tricyclic antidepressants (TCAs), as first-line or augmenting drugs, were widely used to treat CP conditions, including headache, migraine, neuropathic pain, chronic low back pain, fibromyalgia, chronic widespread pain, and abdominal and gastrointestinal pain (68). Amitriptyline is the most useful TCA for various pain syndromes (68). Meanwhile, TCAs can also improve sleep quality (69). Recently, a systematic review and network meta-analysis showed that amitriptyline has higher efficacy for improving sleep, fatigue, and overall quality of life in patients with fibromyalgia (70).

##### 2.5.1.2. The effect of drugs used in sleep management on chronic pain

###### 2.5.1.2.1. Benzodiazepine receptor agonists (BzRAs)

BZRAs are the most well-known extensively prescribed medication to treat sleeping disorders used as adjuvant therapy for pain management. Recently, a narrative review has suggested that BZRAs have analgesic benefits for burning mouth syndrome and stiff person syndrome and for treating co-occurring insomnia and anxiety disorders for short periods of time (2–4 weeks) in CP management (71). Special attention should be required because co-prescribing of BZRAs with opioids for CP and insomnia is common in clinical practice (72). In 2016, the U.S. Food and Drug Administration (FDA) issued a strong official strong warning for the co-usage of opioids and benzodiazepines due to an increased risk of overdose deaths.^1^ Meanwhile, the US Centers for Disease Control and Prevention (CDC) also released the Guideline for prescribing opioids for chronic pain in March 2016, and explicitly stated that “clinicians should avoid prescribing opioid pain medication and benzodiazepines concurrently whenever possible” (73). For example, BZRAs combined with buprenorphine can produce serious respiratory depression (74).

###### 2.5.1.2.2. Melatonin

Melatonin has a significant role in regulating the sleep–wake cycle and inhibits arousal signals. The existing evidence shows that melatonin can reduce the CP (75). A randomized, double-blinded, controlled trial has demonstrated that melatonin as an adjunct therapy to pregabalin reduced the pain score and pain-related sleep interference scores in patients with painful diabetic neuropathy (PDN) (76).

###### 2.5.1.2.3. Suvorexant

Suvorexant is a selective, dual orexin receptor antagonist approved in the USA and Japan for the treatment of insomnia (77). A double-blind, crossover study has shown that suvorexant improved sleep time and reduced next-day pain sensitivity in patients with fibromyalgia (78).

#### 2.5.2. Non-pharmacological approaches

##### 2.5.2.1. Cognitive-behavioral therapy

Cognitive behavioural therapy (CBT) is also the most commonly used psychological approach to treat CP (79). Meanwhile, CBT is recommended as a non-pharmacologic multimodal combination of treatments for coping with sleep problems and as a first-line therapy for insomnia (80, 81). A pilot trial has suggested that hybrid CBT is effective in reducing headache days and insomnia symptoms, which are maintained for 3 months, in youth with co-occurring chronic migraine and insomnia (82). A randomized controlled trial (RCT) study has found that CBT for insomnia (CBT-I) was superior to CBT for pain (CBT-P), which could improve self-reported WASO, SE, SQ, and dysfunctional beliefs and attitudes about sleep (83). Likewise, a systematic review and network meta-analysis has also confirmed that CBT-I might be the most effective treatment option for individuals with comorbid insomnia and CP (84).

##### 2.5.2.2. Complementary and alternative approaches

Althogh not recommended by current guidelines, many alternative and complementary therapies are popular worldwide and have been increasingly studied to treat CP and sleep disorders, including music therapy, aromatherapy, massage, and acupuncture (85, 86). Among these therapies, acupuncture is widely known as one of the common forms to alleviate pain and improve sleep (87, 88). For example, acupuncture treatment can reduce pain pressure thresholds and improve sleep disturbances in patients with fibromyalgia (89). A systematic review and meta-analysis has also shown that acupuncture can relieve pain and improve sleep quality in patients with CP-related insomnia (90).

## 3. Research on the underlying mechanism of chronic pain and sleep disorder relationship

Although CP accompanied by sleep disorders is commonly encountered clinically, our knowledge about the basic neurochemical mechanisms remains rudimentary. Here, we review the majority of recent findings on the perspective of neurochemical mechanisms.

### 3.1. Monoaminergic system

Serotonin, dopamine, and norepinephrine are neurotransmitters of the monoaminergic system, which are involved in the regulation of the endogenous pain system and the sleep–wake system (91, 92). Hyperactivity of monoamine neurons is one of the mechanisms of sleep disorders secondary to CP (93, 94).

#### 3.1.1. Serotonin

Serotonin (5-hydroxytryptamine, 5-HT), the main effector of the serotonergic system, exhibits its effect by activating different receptor subtypes. Neuropathic pain can accelerate the activity of 5-HTergic neurons of the dorsal raphe nucleus (DRN), and activated 5-HTergic neurons produce a significant increase in wakefulness and a significant decrease in NREM sleep in a sciatic nerve ligation (CCI) mouse model (93). Mirtazapine, a noradrenergic and specific serotonergic antidepressant, normalizes the reduction in sleep time and fragmented sleep, regaining the sleep depth at sleep onset in the CP state, and increases the percentage of REM sleep in nerve-ligated mice (95). The selective 5-hydroxy-tryptamine 2A (5-HT2A) antagonist (MDL 100907), administered by intraperitoneal injection, significantly reduces the wake time and improves non-REM sleep time in a CCI mouse model but is not associated with pain relief (96).

#### 3.1.2. Dopamine

Dopamine is a neurotransmitter and neuromodulator that can target dopamine neurons to cause inhibitory or excitatory effects (97). A cross-sectional study has shown that 32.6% of migraine patients have dopaminergic symptoms, including yawning, somnolence, and nausea, and suggested that dopaminergic system modulation should be carefully considered (98). The mesolimbic DA system plays an important role in CP, insomnia, and depression, and the three frequently co-occur (99). Intraperitoneal injection of Levo-tetrahydropalmatine (l-THP), a partial agonist for dopamine D1 receptors (D1R) and an antagonist of D2R, exerts analgesic effects by agonism of D1R and antagonism of D2R, and the antagonism of D2R mediates the hypnotic effect of l-THP in a partial sciatic nerve ligation (PSNL) mouse model (100).

#### 3.1.3. Norepinephrine

Norepinephrine (NE) is an important neurotransmitter in the central nervous system. The locus coeruleus (LC) is the primary source of NE in the brain. The LC-spinal cord noradrenergic pathway is one of the most important pain inhibitory pathways that release norepinephrine (NE) to inhibit the ascending of pain signals (101). NE level increase in the brain is responsible for many of the sleep loss-associated symptoms (102). The activity of LC-NE determines the likelihood of sensory-evoked awakenings (103). However, there is no direct evidence showing that NE is involved in CP-induced sleep disorders.

### 3.2. Adenosine

Adenosine is a purine nucleoside that exerts a broad range of biological effects by binding to adenosine receptors (ARs). Adenosine exerts the analgesic effect primarily via the activation of A1AR located at peripheral, spinal, and supraspinal sites (104). On the other hand, adenosine plays an important role in integrating light and sleep signaling by the activation of A1/A2AR for the regulation of circadian timing (105). Animal experiments have confirmed that adenosinergic signaling regulates sleep-pain interactions. Systemic administration of the nonselective adenosine receptor antagonist caffeine prevents the sleep deprivation-induced increase in postoperative hypersensitivity, while microinjection of the adenosine A2A receptor antagonist into the median preoptic nucleus blocks the increase in surgical pain levels and duration caused by prior sleep deprivation and eliminats the thermal hyperalgesia induced by sleep deprivation in naive rats (106).

### 3.3. Melatonin

Melatonin is a neuroendocrine hormone, mainly synthesized and secreted by the pineal gland, which has a wide range of physiological functions, including regulation of circadian rhythms, enhancement of immune function, improvement of sleep, and pain (107). Sleep deprivation enhances microglial activation and aggravates neuropathic pain by suppressing melatonin secretion in a CCI rat model (108). Melatonin receptor agonist (piromelatine) significantly prolongs thermal and mechanical latencies and increases NREM sleep in a partial sciatic nerve ligation (PSL) mouse model (109). Furthermore, The antinociceptive effect of piromelatine is mediated by melatonin, opioid, and 5HT1A receptors; while the hypnotic effect of piromelatine is mediated by melatonin receptors (109).

### 3.4. Gamma-aminobutyric acid and glutamate

Gamma-aminobutyric acid (GABA) and glutamate (Glu) are major inhibitory and excitatory neurotransmitters, respectively. A proton magnetic resonance spectroscopy study has found that patients with chronic migraine had significantly lower levels of GABA in the dentate nucleus (DN) and higher levels of Glu in the periaqueductal gray (PAG), and higher GABA levels in the PAG were significantly associated with poorer sleep quality in all patients with migraine (110). The extracellular GABA concentration deceases in the cingulate cortex, which is associated with sleep disturbance in neuropathic pain mice (111).

## 4. Discussion

Sleep disorders secondary to chronic pain are a very common phenomenon. There is considerable evidence showing a reciprocal association between pain and sleep, especially in CP. CP results in insufficient sleep time and quality, which in turn increases pain sensitivity and severely compromises the pain management and treatment outcomes of patients. CP can affect sleep in terms of sleep time, sleep structure, sleep depth, etc., resulting in reduced sleep time, sleep structure disorder, sleep fragmentation, and reduced sleep depth. Sleep fragmentation in synergy with CP may lead to prolonged and exacerbated allodynia. Moreover, sleep quality can be a predictor of next-day pain, and short-term improved sleep can contribute to long-term clinical benefits in CP patients. However, sleep assessment is not performed in patients with CP in routine clinical practice (112). Thus, evaluation of sleep is recommended for investigating patients with CP. On the one hand, this may assist in the selection of rational therapeutic strategies for clinicians in clinical practice. On the other hand, this can also help further understand sleep characteristics and sleep disorders mechanisms, which is beneficial for the development of new potential therapeutic agents and treatment strategies in CP patients with comorbid sleep disorders. Of course, we should note that the sleep disorders and CP are “equal” in some cases. Painful and nonpainful somatic symptoms, including sleep disturbance, appetite disturbance, and fatigue or loss of energy, essentially characterize clinical states of depressive mood (113). It is worth to mention, as in the case even “adequate pain management” will not improve insomnia being not just a secondary to CP.

At present, the first choice for CP control is still drug therapy; however, there is still a lack of ideal drugs that are proven to be effective in both aspects. In CP, many drugs can only relieve pain but cannot improve sleep disorders or have hypnotic effects but cannot solve the pain problem. Therefore, it is of great significance to elucidate the underlying mechanisms of the interaction between CP and sleep disorders and seek new treatments. Special attention should be given to the possibility of a risk of drug–drug interaction when using two types of drugs targeting pain and sleep simultaneously. Therefore, more clinical data and basic research are required. At the same time, the available clinical evidence has suggested that nonpharmacologic therapy (CBT and complementary and alternative therapies) has certain therapeutic effects on pain and sleep and should receive more attention.

**Figure 1:**
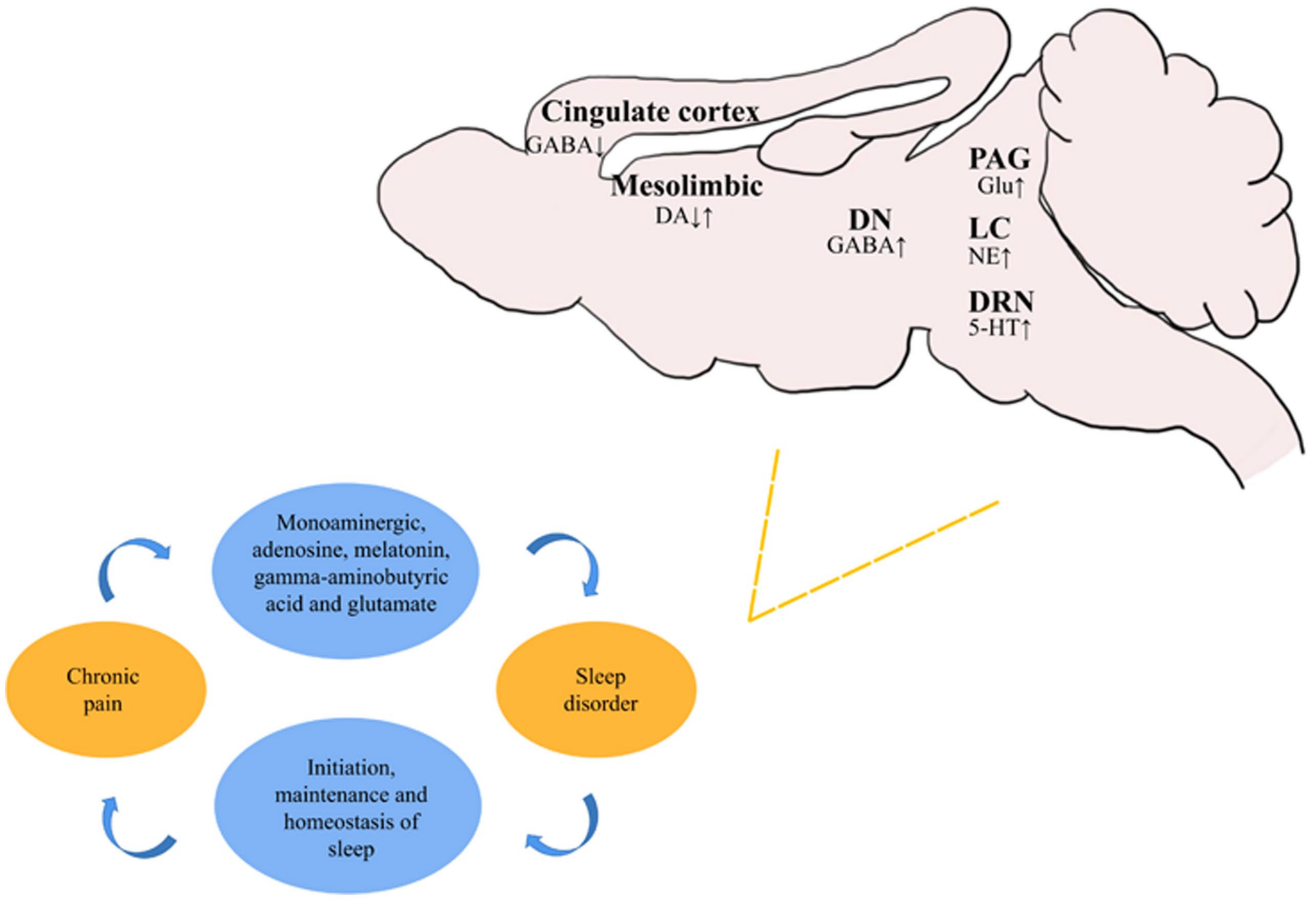
Recent studies on the changes of different neurons in various brain regions during sleep disorders secondary to chronic pain. ↑ represents the activation or activity enhancement of neurons, and ↓ represents the inhibition or activity reduction of neurons.

There is a considerable amount of research on underlying mechanisms for the development of CP and sleep disorders. Neurotransmitters, such as melatonin, cortisol, norepinephrine, and dopamine, are involved in the control of the circadian clock, as well as the regulation of pain perception and pain. CP-induced sleep disorders are closely related to the monoaminergic, adenosine, histamine, melatonin, GABAergic, and orexinergic systems. The initiation and maintenance of sleep, as well as sleep homeostasis, are regulated by complex pathways in these systems (Figure 1). Such results clearly demonstrate that control over the neurophysiological mechanisms medicating pain may provide an important route for treating pain and sleep disorder behavior.

This study has several limitations. As a narrative review, some relevant articles may have not been included. We only focused on neurochemical mechanisms. Other pathologic mechanisms in neuroplasticity and neural circuitry on sleep disorders secondary to CP are also very important, which were not involved here. Furthermore, sleep problems in persons with CP were a complicated phenomenon involving not only physiological but also psychological and social factors.

Our review aimed to raise awareness for both psychiatric and non-psychiatric practitioners about the importance of sleep disorders secondary to chronic pain. The presence of sleep disorders in CP can aggravate the pain, as well as seriously affect the quality of life of patients. In terms of treatment, CBT is the best non-pharmacological intervention, while pharmacological treatments require further in-depth research. The research on sleep disorders secondary to CP is still in its infancy, and further elucidation of the underlying mechanisms of the interaction between CP and sleep disorders is crucial for developing more effective therapeutic strategies to improve pain and sleep.

## Author contributions

KW and JZ directed the project and revised the manuscript. KW, LD, and XY designed research. LD, XY, RH, and XD were involved in bibliographic research and data collection. LD and XY wrote the manuscript. LD, XY, RH, XD, JZ, and KW discussed the results and commented on the manuscript. All authors contributed to the article and approved the submitted version.

## Funding

This work was financially supported by the National Natural Science Foundation of China (81973940), Shanghai Clinical Research Center for Acupuncture and Moxibustion (20MC1920500), and Shanghai Municipal Commission of Health and Family Planning (ZY(2021-2023)-0208).

## Conflict of interest

The authors declare that the research was conducted in the absence of any commercial or financial relationships that could be construed as a potential conflict of interest.

## Publisher’s note

All claims expressed in this article are solely those of the authors and do not necessarily represent those of their affiliated organizations, or those of the publisher, the editors and the reviewers. Any product that may be evaluated in this article, or claim that may be made by its manufacturer, is not guaranteed or endorsed by the publisher.

## Footnotes

^1^ https://www.fda.gov/news-events/press-announcements/fda-requires-strong-warnings-opioid-analgesics-prescription-opioid-cough-products-and-benzodiazepine
